# Association Between Sleep Quality and Postcolonoscopy Pain

**DOI:** 10.5152/tjg.2026.26044

**Published:** 2026-05-03

**Authors:** Haci Bolat, Tugba Tolu, Haluk Tarik Kani

**Affiliations:** 1 Department of General Surgery, Niğde Ömer Halisdemir University Faculty of Medicine, Niğde, Türkiye; 2 Department of Gastroenterology, Marmara University School of Medicine, İstanbul, Türkiye; 3 Marmara University Institute of Gastroenterology, İstanbul, Türkiye

**Keywords:** Brain–gut axis, colonoscopy, patient satisfaction, postprocedural pain, psychosomatic factors, sleep quality

## Abstract

**Background/Aims:**

Postcolonoscopy pain remains a key determinant of patient satisfaction and willingness to undergo repeat procedures. Patient-related factors influencing postprocedural pain are not fully understood, and the role of sleep quality has not been systematically elucidated. This study aimed to investigate the association between sleep quality and postcolonoscopy abdominal pain in a real-world cohort.

**Materials and Methods:**

This single-center, prospective observational cohort study included adults aged 18-80 years undergoing diagnostic or surveillance colonoscopy. All procedures were performed by a single experienced endoscopist under standardized conditions. Psychological and sleep-related parameters were assessed using validated instruments, including the Beck Depression Inventory, State–Trait Anxiety Inventory, Van Dream Anxiety Scale, and Pittsburgh Sleep Quality Index. Pain intensity was measured using a Visual Analog Scale at 0 minutes, 30 minutes, 6 hours, and 24 hours after the procedure. Associations between psychometric measures and pain scores were analyzed using Spearman’s correlation.

**Results:**

A total of 103 patients were included (mean age, 54.7 ± 12.4 years; 45.6% female). Postcolonoscopy pain was most prominent 0 minutes after the procedure and decreased progressively over time. Poor sleep quality was significantly associated with higher pain intensity at 6 hours after colonoscopy (*r* = 0.239, *P* = .015). Anxiety and depressive symptom severity scores were not associated with pain intensity; however, a prior clinical diagnosis of depression was associated with higher postprocedural pain scores. Higher pain scores were also observed in patients with incomplete procedures, gastrointestinal symptoms, and postprocedural dissatisfaction (*P* < .05).

**Conclusion:**

Sleep quality may represent an important patient-related factor influencing early postcolonoscopy pain perception. Consideration of sleep and psychosocial factors alongside routine clinical evaluation may help improve the understanding of postprocedural pain variability and enhance patient-centered endoscopy care.

**Main Points**

- Postcolonoscopy pain is common at 0 minutes after the procedure but largely resolves within 24 hours.
- Poor sleep quality is associated with higher abdominal pain intensity at 6 hours after colonoscopy.
- Anxiety and depressive symptom severity scores are not associated with postprocedural pain intensity; however, a prior clinical diagnosis of depression is linked to higher pain scores.
- Patient-related psychosocial factors, particularly sleep quality, may contribute to early postcolonoscopy pain variability.

## Introduction

Colonoscopy remains a key diagnostic and therapeutic procedure in the management of gastrointestinal diseases. Recent advances in endoscopic technology have improved its effectiveness in the early detection and treatment of gastrointestinal pathology.^1^ Nevertheless, patient-related barriers to colonoscopy persist. Preprocedural anxiety, which is often driven by concerns about pain, embarrassment, or procedural discomfort, remains common.^2^^-^^4^ In addition, postprocedural pain may negatively influence patients’ willingness to undergo repeat colonoscopic examinations in the future.^5^^,^^6^

Despite ongoing research, no definitive strategies exist to prevent postcolonoscopy pain. Studies on functional gastrointestinal disorders have demonstrated considerable interindividual variability in pain perception and thresholds, suggesting that patient-related factors may play an important role in postprocedural pain experiences.^5^^,^^7^

A bidirectional relationship exists between the brain and the gut, mediated by the central, autonomic, and enteric nervous systems. Within this gut–brain axis, circadian rhythms play an important regulatory role in gastrointestinal physiology, and their disruption has been shown to adversely affect gastrointestinal motility, visceral sensitivity, and mucosal function.^8^^-^^11^

Melatonin, a key regulator of circadian rhythm, has been shown to influence gastrointestinal motility, visceral sensitivity, and pain modulation. Experimental and clinical studies have demonstrated that melatonin supplementation can reduce abdominal pain and improve bowel symptoms in functional gastrointestinal disorders.^12^ Importantly, sleep disturbances have also been associated with rectal hypersensitivity, a physiological marker of increased visceral pain perception within the gut–brain axis.^13^

These observations suggest that disruptions in neural regulatory pathways within the gut–brain axis may amplify gastrointestinal pain perception. This raises the possibility that sleep disturbances could exacerbate postcolonoscopic pain responses.

Although psychosomatic and lifestyle-related factors have been studied in chronic gastrointestinal disorders, the role of sleep disturbance in acute postprocedural pain remains poorly understood. To the best of the authors’ knowledge, no previous study has directly examined the relationship between sleep quality and abdominal pain after colonoscopy. Therefore, the aim of this study was to evaluate the association between sleep quality and postcolonoscopy abdominal pain in a real-world clinical cohort.

## Materials and Methods

### Study Design and Setting

This single-center prospective observational cohort included patients aged 18-80 years scheduled for diagnostic or surveillance colonoscopy between July 2021 and December 2021. Exclusion criteria included shift work, prior colorectal surgery, chronic obstructive pulmonary disease, refusal to participate, and age outside the predefined range. All colonoscopies were performed by a single experienced endoscopist to ensure procedural standardization.

### Colonoscopy Procedure and Data Collection

All patients underwent bowel preparation with a polyethylene glycol–based solution prior to colonoscopy. Blood pressure, heart rate, and oxygen saturation levels were monitored at baseline and continuously throughout the procedure. Room air was used for colonic insufflation. Sedation was administered intravenously with midazolam, and the dosage was individualized by the endoscopist based on each patient’s clinical condition and medical history. Colonoscopy was considered successful when the cecum was reached and visualized. Procedures in which cecal intubation was not achieved were classified as incomplete colonoscopies. Difficult colonoscopy was defined, according to American Society of Gastroinstestinal Endoscopy (ASGE) and European Society of Gastrointestinal Endoscopy (ESGE) quality indicators, as procedures requiring more than 15 minutes to reach the cecum or requiring repeated ancillary maneuvers (such as loop reduction, external abdominal pressure, patient repositioning, or additional sedation) to achieve completion.^14^ Ileal intubation was recorded upon entering and visualizing the terminal ileum. Patient dissatisfaction was recorded as a binary variable (satisfied vs dissatisfied) based on the patient’s self-report 0 minutes after the procedure, consistent with established methodology in prior studies assessing endoscopy quality.^15^

For all patients, sociodemographic characteristics, body mass index (BMI), history of abdominal surgery, pre- and postcolonoscopy abdominal circumference, total procedure time, ileal intubation status, and midazolam dose were recorded.

### Psychological and Sleep Assessments

Patients’ levels of anxiety and depression were evaluated using the Beck Depression Inventory (BDI), a 21-item self-reported questionnaire in which each item is scored on a scale from 0 to 3, with higher scores indicating greater severity of depressive symptoms.^16^ The validated Turkish version of the scale was established in 2008 based on psychometric evaluation using Diagnostic and Statistical Manual of Mental Disorders - IV (DSM-IV) criteria. For the Turkish population, cutoff scores were defined as follows: 0 to 12 (minimal depression), 13 to 18 (mild depression), 19 to 28 (moderate depression), and 29 to 63 (severe depression).^17^ In addition, prior clinical diagnosis of depression was recorded as a binary variable based on documented patient history.

Sleep quality disturbances associated with anxiety were assessed using the Pittsburgh Sleep Quality Index (PSQI),^18^ a 19-item scale that assesses sleep quality and comprises 7 components. Each of the 7 components is scored on a scale from 0 to 3. A global PSQI score of 5 or higher indicates poor sleep quality. The Turkish version of the PSQI was validated in 1996.^19^

Sleep-related anxiety was evaluated using the Van Dream Anxiety Scale (VDAS),^20^ a scale developed in Türkiye to assess nightmare frequency and dream-related anxiety. It consists of 17 items: 12 assess dream-related anxiety, 4 are clinical questions, and 1 assesses autonomic symptoms. The total score ranges from 0 to 52. Although a definitive cutoff value has not been established, the original validation study reported mean control group scores of 8 to 12, with scores of 20 or higher generally associated with clinically relevant dream anxiety.^21^^,^^22^

### Pain Assessment

Postcolonoscopy pain was assessed using a Visual Analog Scale (VAS) at 0 minutes, 30 minutes, 6 hours, and 24 hours after the procedure. The VAS consisted of a 10-cm horizontal line ranging from 0 (no pain) to 10 (worst possible pain).^23^

Patients completed self-administered questionnaires, with clinician assistance when necessary. Pain intensity was assessed using the same VAS at 0 minutes, 30 minutes, 6 hours, and 24 hours after colonoscopy. The 0- and 30-minute assessments were performed in the endoscopy unit, whereas the 6- and 24-hour assessments were conducted through structured telephone interviews. All responses were recorded in a study database.

### Statistical Analysis

Continuous variables are expressed as mean ± SD for normally distributed data or as median (minimum-maximum) for non-normally distributed data, whereas categorical variables are presented as counts and percentages. Group comparisons were performed using the independent samples *t*-test or the Mann–Whitney *U-*test for continuous variables and the chi-square test or Fisher’s exact test for categorical variables. For comparisons across more than 2 groups, 1-way analysis of variance was used for normally distributed variables and the Kruskal–Wallis test for non-normally distributed variables. Correlations between pain scores and psychological or sleep-related measures were assessed using Spearman’s rank correlation coefficients. Additional exploratory analyses were performed to evaluate associations between colonoscopy outcomes and clinical or demographic variables. A 2-sided *P*-value < .05 was considered statistically significant.

**Ethics Approval**

The study protocol was approved by the Institutional Ethics Committee of Niğde Ömer Halisdemir University, and all procedures were conducted in accordance with the principles of the Declaration of Helsinki (Approval Date: July 8, 2021, Approval number: 2021/71). Written informed consent was obtained from all participants before study enrollment.

## Results

A total of 103 patients were enrolled in the study. The mean age was 54.6 ± 12.4 years, and 47 patients (45.6%) were female. Previous colonoscopy had been performed in 24 patients (23.3%), and 12 (11.7%) had a history of abdominal surgery. Diabetes mellitus was present in 10 patients (9.7%), and 4 (3.9%) had a prior diagnosis of depression. Baseline demographic and clinical characteristics are presented in Table 1.

The most common indication for colonoscopy was constipation (n = 28, 27.2%), followed by surveillance (n = 25, 24.3%) and hematochezia (n = 11, 10.7%). Biopsy and polypectomy were performed in 13 (12.6%) and 12 patients (11.7%), respectively. Colonoscopy could not be completed in 6 patients (5.8%), ileal intubation was achieved in 17 patients (16.5%), and difficult colonoscopy was recorded in 9 patients (8.7%). Bowel cleansing was insufficient in 29 patients (28.2%). One procedure-related complication occurred during colonoscopy (arrhythmia). Procedural indications and findings are summarized in Table 2.

**Figure 1:**
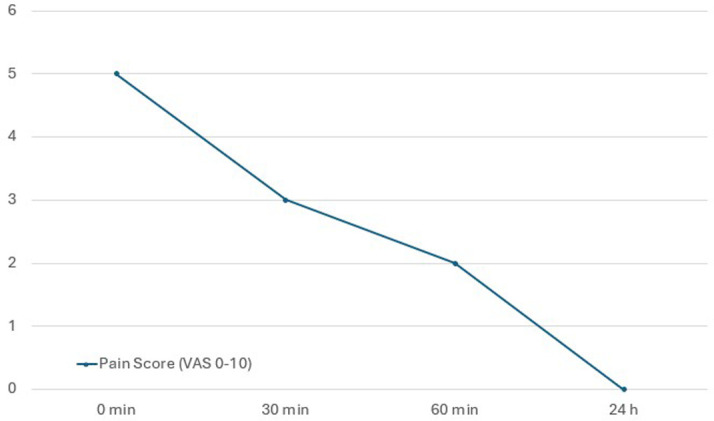
Median postcolonoscopy abdominal pain scores measured at 0 minutes, 30 minutes, 60 minutes, and 24 hours after the procedure. VAS, Visual Analog Scale.

Postcolonoscopy pain was common in the early postprocedural period and decreased progressively over time. Median VAS scores were 5 (range, 0-8) at 0 minutes after the procedure, 3 (0-7) at 30 minutes, 2 (0-6) at 60 minutes, and 0 (0-5) at 24 hours (Figure 1).

**Figure 2:**
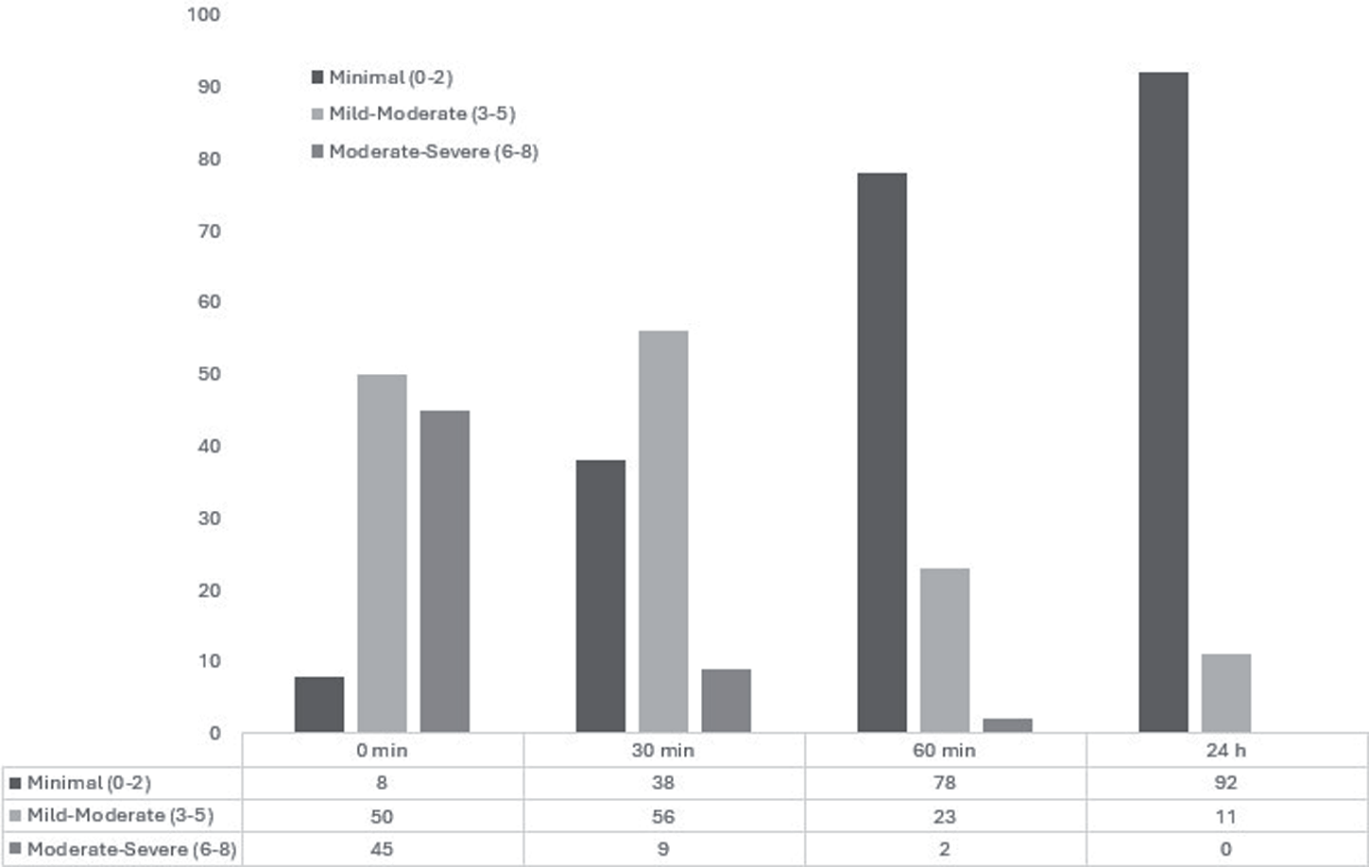
Distribution of post-colonoscopy abdominal pain severity over time according to VAS categories. VAS, Visual Analog Scale.

At 0 minutes, 45 patients (43.7%) experienced moderate-to-severe pain; this proportion decreased to 9 (8.7%) at 30 minutes, 2 (1.9%) at 60 minutes, and 0 at 24 hours. Conversely, the proportion of patients reporting minimal pain increased from 8 (7.8%) immediately after the procedure to 92 (89.3%) at 24 hours (Figure 2).

Psychometric and sleep-related scores indicated a generally low-to-moderate symptom burden in the cohort. Median scores were 7 (range, 0-42) for the BDI, 39 (range, 20-66) for the State-Trait Anxiety Inventory (STAI)-1, and 44 (range, 24-69) for the STAI-2. Median scores were 6 (range, 2-19) for the PSQI and 5 (range, 0-54) for the Dream Anxiety Scale. Detailed categorical distributions of these measures are presented in Supplementary Table 1.

Correlation analysis showed a weak but statistically significant positive correlation between PSQI and VAS scores at 6 hours (*r* = 0.239, *P* = .015). No significant correlations were observed between pain scores at 0 minutes, 30 minutes, or 24 hours and BDI, STAI-1, STAI-2, PSQI, or Dream Anxiety Scale scores. Significant positive correlations were observed among several psychological variables, including BDI with STAI-1, STAI-2, and Dream Anxiety Scale scores; STAI-1 and STAI-2 with Dream Anxiety Scale scores; and PSQI with Dream Anxiety Scale scores (Table 3).

In univariate analyses, higher body weight and BMI were associated with difficult colonoscopy (*P* = .023 and *P* = .002, respectively). Patient dissatisfaction was consistently associated with higher pain scores at all postprocedural time points (0 minutes, 30 minutes, 6 hours, and 24 hours; all *P* ≤ .002). Diabetes mellitus was more frequent among patients with difficult colonoscopy (*P* = .005), whereas prior diagnosis of depression and prior abdominal surgery were more frequent among patients with incomplete procedures (both *P* = .016). Incomplete colonoscopy was also associated with higher pain severity at 6 hours (*P* = .014; Supplementary Table 2). Colonoscopy history was associated with difficult colonoscopy (*P* < .001) but was not associated with incomplete colonoscopy, ileal intubation, or patient dissatisfaction. Detailed exploratory analyses are presented in Supplementary Tables 3A and 3B.

## Discussion

In this prospective observational cohort study, postcolonoscopy pain was primarily early in onset and self-limited. Pain intensity was highest 0 minutes after the procedure and was largely resolved within the first postprocedural day.

Patients with a prior diagnosis of depression and those with poorer sleep quality reported higher postprocedural pain. Notably, although a prior diagnosis of depression was associated with increased pain perception, BDI scores were not. This discrepancy may reflect indirect pathways, such as concurrent anxiety or poor sleep quality, rather than the severity of depressive symptoms alone. Although anxiety measures (STAI-1 and STAI-2) and dream-related anxiety (VDAS) were not directly associated with postcolonoscopy pain, these psychological domains were moderately to strongly correlated with each other (*r* = 0.35-0.45, *P* < .001). BDI scores also correlated positively with both STAI and dream-related anxiety.

Impaired sleep quality, assessed using the PSQI, was the only parameter significantly associated with pain intensity at 6 hours after the procedure (*r* = 0.239, *P* = .015), reinforcing the potential role of sleep disturbances in modulating postprocedural pain perception.

To date, no systematic study has specifically examined the association between sleep quality and postprocedural gastrointestinal pain. Lei et al^24^ reported in a large cross-sectional cohort that sleep disturbance was significantly associated with functional gastrointestinal symptoms, including dyspepsia and irritable bowel syndrome. Experimental and clinical studies have also consistently demonstrated that sleep disruption increases pain sensitivity and impairs central pain modulation.^25^^,^^26^ Sleep deprivation has also been linked to enhanced visceral hypersensitivity through alterations in brain–gut axis signaling.^27^^,^^28^ However, whether impaired sleep influences acute pain perception after endoscopic procedures has remained largely unclear. The findings of this study extend this evidence by demonstrating that impaired sleep quality was modestly but significantly associated with higher pain intensity 6 hours after colonoscopy (*r* = 0.239, *P* = .015), consistent with previous reports linking poor sleep to increased pain sensitivity.^29^^,^^30^ Although the observed correlation was relatively weak, the direction of the association is consistent with the growing body of literature linking poor sleep quality to increased pain sensitivity and altered gastrointestinal sensory processing. To the authors’ knowledge, the current study represents one of the first clinical investigations exploring the relationship between sleep quality and postcolonoscopy pain.

Additionally, the findings complement those of the prospective study by Lee et al,^5^ which investigated predictors of postcolonoscopy pain in a large screening cohort. In that study, female sex and longer procedure duration were significantly associated with increased pain, whereas age, BMI, sedation, and bowel preparation were not. In this cohort, higher body weight was associated with difficult colonoscopy, which may contribute to increased procedural manipulation and subsequent discomfort. These differences may partly reflect variations in study populations. Although the study by Lee et al focused primarily on asymptomatic individuals undergoing screening colonoscopy, this cohort included both symptomatic and asymptomatic patients. This broader clinical spectrum may introduce greater heterogeneity in psychological burden and pain perception, potentially influencing postprocedural pain responses.

In line with the observations of the current study, Zaczek et al^31^ also reported associations between affective symptoms and pain perception. However, their study was limited by a relatively small sample size and considerable attrition, which may have reduced statistical power. Despite these limitations, their findings are broadly consistent with the findings of this study. Importantly, this work expands this perspective by incorporating sleep quality, demonstrating that impaired sleep was modestly but significantly associated with pain intensity 6 hours after colonoscopy. Taken together, these findings suggest that while procedural and demographic factors may play a dominant role in screening populations, psychosomatic dimensions (including trait anxiety, dream-related distress, and sleep quality) may contribute more substantially to pain perception in real-world symptomatic cohorts. This underscores the importance of integrating psychosocial assessment into periprocedural evaluation to optimize patient experience and minimize postcolonoscopy discomfort.

The current study also demonstrated that patients with incomplete procedures, those presenting with gastrointestinal symptoms, and those reporting postprocedural dissatisfaction had higher VAS scores at both 6 and 24 hours (*P* < .05). These findings are consistent with previous literature showing that prolonged or technically difficult procedures are associated with greater postprocedural discomfort^5^ and that pre-existing gastrointestinal symptoms may reduce pain thresholds through mechanisms of visceral hypersensitivity.^32^ Collectively, these observations suggest that both procedural complexity and patient-related factors contribute to the variability in postcolonoscopy pain experience.

Another important observation of this study was the strong association between patient dissatisfaction and higher VAS scores. This relationship may be bidirectional: inadequate pain control is a well-established predictor of poor satisfaction, whereas dissatisfaction itself may exacerbate the subjective perception of pain through psychological mechanisms such as increased attentional focus and negative affect. Prior studies have shown that patient satisfaction with endoscopic procedures is closely associated with pain and comfort levels during and after the procedure.^15^ These findings highlight the potential value of incorporating patient-reported experience measures alongside objective pain assessments to provide a more comprehensive evaluation of procedural outcomes. This observation suggests that postprocedural pain and patient satisfaction should be interpreted as interrelated outcomes rather than independent endpoints.

A major strength of the current study was the use of a standardized colonoscopy protocol, with all procedures performed by a single experienced endoscopist, thereby minimizing interoperator variability. Data were collected prospectively using predefined assessments, ensuring methodological consistency throughout the study period. The cohort demonstrated a high colonoscopy completion rate (94.2%), whereas technically difficult procedures were uncommon (8.7%) and complications were rare (0.9%). This standardized procedural environment provided a relatively homogeneous dataset, allowing for a more reliable evaluation of postcolonoscopy pain outcomes.

Several limitations of this study should be acknowledged. First, the relatively small sample size and the low number of patients reporting severe pain may have limited the statistical power to detect independent predictors of postcolonoscopy pain. Second, early pain assessments performed at 0 and 30 minutes after the procedure may have been influenced by residual sedative effects of midazolam, potentially introducing measurement bias despite the use of a standardized sedation protocol. Third, although data were collected prospectively, the observational design limits the ability to establish causal relationships between psychosomatic factors and pain perception. Future studies with larger and more heterogeneous populations are needed to validate these associations and further clarify the role of sleep quality and psychological factors in postprocedural pain.

In conclusion, these findings suggest that psychosocial factors, particularly sleep quality, may contribute to variability in postcolonoscopy pain perception. These results highlight the potential relevance of incorporating psychological and sleep-related factors into a broader periprocedural assessment. Incorporating patient-reported outcomes, including pain and satisfaction measures, may provide a more comprehensive evaluation of the colonoscopy experience and quality of care.

## Supplementary Materials

Supplementary Material

## Figures and Tables

**Table 1.** Demographic and Clinical Characteristics of Patients

**Variable**	**Value**
Age, mean ± SD (years)	54.6 ± 12.4
Sex, n (%)	
Female	47 (45.6)
Male	56 (54.4)
Height, mean ± SD (cm)	166.7 ± 9.0
Weight, mean ± SD (kg)	76.0 ± 13.0
Body mass index, mean ± SD (kg/m²)	27.4 ± 4.3
Diabetes mellitus, n (%)	10 (9.7)
Depression diagnosis, n (%)	4 (3.9)
History of abdominal surgery, n (%)	12 (11.7)
Previous colonoscopy, n (%)	24 (23.3)
Difficult colonoscopy, n (%)	9 (8.7)
Completed procedure, n (%)	97 (94.2)
Ileal intubation, n (%)	17 (16.5)
Patient dissatisfaction, n (%)	9 (8.7)

Values are presented as mean ± standard deviation for continuous variables and as n (%) for categorical variables.

**Table 2.** Indications and Findings of Colonoscopy Procedures

**Variable**	**n (%)**
Indication for colonoscopy	
Constipation	28 (27.2)
Surveillance	25 (24.3)
Polypectomy	7 (6.8)
Abdominal pain	10 (9.7)
Diarrhea	5 (4.9)
Hematochezia	11 (10.7)
Bloating	6 (5.8)
Anemia	9 (8.7)
Other	2 (1.9)
Endoscopic findings	
Tumor	4 (3.9)
Polyp	17 (16.5)
Diverticulum	1 (1.0)
Other	6 (5.8)
Additional intervention	
Biopsy	13 (12.6)
Segmental biopsy	3 (2.9)
Polypectomy	12 (11.7)
Other	3 (2.9)

**Table 3.** Correlation Analyses between Psychological-Sleep Parameters and Pain Scores

**Variable 1**	**Variable 2**	**Correlation Coefficient**	***P***
BDI	STAI-1 (state anxiety)	0.400	**<.001**
BDI	STAI-2 (trait anxiety)	0.411	**<.001**
BDI	Dream anxiety (VDAS)	0.450	**<.001**
STAI-1	Dream anxiety (VDAS)	0.408	**<.001**
STAI-2	Dream anxiety (VDAS)	0.354	**<.001**
PSQI	Dream anxiety (VDAS)	0.352	**<.001**
PSQI	VAS, 6 hours	0.239	**.015**

BDI, Beck Depression Inventory; PSQI, Pittsburgh Sleep Quality Index; STAI, State-Trait Anxiety Inventory; VAS, Visual Analog Scale; VDAS, Van Dream Anxiety Scale.

## Data Availability

The data that support the findings of this study are available on request from the corresponding author.
