# Supplementary material for: Association Between Sleep Quality and Postcolonoscopy Pain
Source: Turk J Gastroenterol. 2026 May 3;37(8):839–46. doi: 10.5152/tjg.2026.26044 (PMC13455510; doi:10.5152/tjg.2026.26044)
Supplement: Supplementary file 1 [file supplementary_material.pdf]

Supplementary Table 1. Pain and psychometric characteristics of the study cohort

Variable	Value
Psychometric scores, median (range)	
Beck Depression Inventory	7 (0–42)
STAI-1	39 (20–66)
STAI-2	44 (24–69)
Pittsburgh Sleep Quality Index	6 (2–19)
Dream Anxiety Scale	5 (0–54)
Beck Depression Inventory categories, n (%)	
Minimal (0–9)	68 (66.0)
Mild (10–16)	24 (23.3)
Moderate (17–29)	6 (5.8)
Severe (30–63)	5 (4.9)
STAI-1 categories, n (%)	
None (0–20)	53 (51.5)
Mild (20–39)	48 (46.6)
Moderate (40–59)	2 (1.9)
Severe (≥60)	0 (0.0)
STAI-2 categories, n (%)	
None (0–20)	38 (36.9)
Mild (20–39)	60 (58.3)
Moderate (40–59)	5 (4.9)
Severe (≥60)	0 (0.0)
Pittsburgh Sleep Quality Index categories, n (%)	
Good sleep (0–5)	44 (42.7)
Poor sleep (6–10)	40 (38.8)
Very poor sleep (>10)	19 (18.4)
Dream Anxiety Scale categories, n (%)	
Low anxiety (0–13)	75 (72.8)
Moderate anxiety (14–21)	15 (14.6)
High anxiety (≥22)	13 (12.6)

Supplementary Table 2. Clinical and procedural factors associated with post-colonoscopy pain severity at 6th hours

Variable	Minimal pain n (%)	Mild–moderate pain n (%)	Moderate–severe pain n (%)	*P*
Female sex	35 (44.9)	11 (47.8)	1 (50.0)	.962
Diabetes mellitus	7 (9.0)	3 (13.0)	0	.758
Previous abdominal surgery	7 (9.0)	4 (17.4)	0	.458
Prior colonoscopy	19 (24.4)	4 (17.4)	1 (50.0)	.523
Difficult colonoscopy	7 (9.0)	2 (8.7)	0	.906
Incomplete colonoscopy	5 (6.4)	0	1 (50.0)	.014
Ileal intubation	13 (16.7)	4 (17.4)	0	.815
Bowel preparation adequate	59 (75.6)	14 (60.9)	1 (50.0)	.302

Supplementary Table 3A. Comparison of continuous variables according to colonoscopy outcomes

Variable	Difficult colonoscopy (No vs Yes)	*P*	Incomplete procedure (No vs Yes)	*P*	Ileal intubation (No vs Yes)	*P*	Patient dissatisfaction (No vs Yes)	*P*
Age, years	54.3 ± 12.8 vs 59.0 ± 5.9	.275	52.0 ± 6.7 vs 54.8 ± 12.7	.589	55.6 ± 12.0 vs 49.9 ± 13.9	.085	56.9 ± 9.5 vs 54.5 ± 12.7	.577
Height, cm	167.0 ± 9.2 vs 164.4 ± 7.9	.428	167.5 ± 8.4 vs 166.7 ± 9.2	.743	166.2 ± 9.1 vs 169.8 ± 9.1	.137	164.3 ± 5.7 vs 167.0 ± 9.4	.343
Weight, kg	75.3 ± 12.8 vs 85.8 ± 14.8	**.023**	74.2 ± 17.1 vs 76.4 ± 13.1	.890	75.9 ± 13.5 vs 78.2 ± 12.0	.502	75.7 ± 19.6 vs 76.3 ± 12.6	.925
BMI, kg/m²	27.0 ± 4.1 vs 31.8 ± 5.3	**.002**	26.3 ± 5.2 vs 27.5 ± 4.3	.401	27.5 ± 4.6 vs 27.1 ± 3.0	.723	28.1 ± 7.3 vs 27.4 ± 4.0	.793

Continuous variables were compared using the independent samples t-test or Mann–Whitney U test as appropriate.

Supplementary Table 3B. Comparison of categorical variables according to colonoscopy outcomes

Variable	Difficult colonoscopy (n = 9)	*P*	Incomplete procedure (n = 6)	*P*	Ileal intubation (n = 17)	*P*	Patient dissatisfaction (n = 9)	*P*
Female sex, n (%)	5 (55.6)	.532	4 (66.7)	.286	6 (35.3)	.349	4 (44.4)	.940
Diabetes mellitus, n (%)	4 (44.4)	**.005**	1 (16.7)	.467	1 (5.9)	1.000	1 (11.1)	1.000
Prior depression diagnosis, n (%)	1 (11.1)	.310	**2 (33.3)**	**.016**	1 (5.9)	.520	1 (11.1)	.310
Prior abdominal surgery, n (%)	1 (11.1)	.310	**2 (33.3)**	**.016**	1 (5.9)	.520	1 (11.1)	.310
Previous colonoscopy, n (%)	**9 (100)**	**<.001**	2 (33.3)	.622	5 (29.4)	.514	3 (33.3)	.456

Categorical variables were compared using the χ^2 test or Fisher's exact test.
